# Effectiveness of brief interventions as part of the screening, brief intervention and referral to treatment (SBIRT) model for reducing the non-medical use of psychoactive substances: a systematic review protocol

**DOI:** 10.1186/2046-4053-1-22

**Published:** 2012-05-07

**Authors:** Matthew M Young, Adrienne Stevens, Amy Porath-Waller, Tyler Pirie, Chantelle Garritty, Becky Skidmore, Lucy Turner, Cheryl Arratoon, Nancy Haley, Karen Leslie, Rhoda Reardon, Beth Sproule, Jeremy Grimshaw, David Moher

**Affiliations:** 1 Canadian Centre on Substance Abuse (CCSA), 75 Albert Street, Ottawa, ON, K1P 5E7, Canada; 2 Department of Psychology, Carleton University, 1125 Colonel By Drive, Ottawa, ON, K1S 5B6, Canada; 3 Ottawa Hospital Research Institute (OHRI), Ottawa Hospital - General Campus, 501 Smyth Road, Ottawa, ON, K1H 8L6, Canada; 4 Independent Research and Information Consultant, Ottawa, Canada; 5 Département de Pédiatrie, Hôpital Sainte-Justine, 3175 Ch DE LA Cote-Sainte-Cath, Montréal, QC, H3T 1C5, Canada; 6 Direction de Santé Publique de Montréal, Faculté de Médecine, Université de Montréal, Montréal, QC, H3C 3J7, Canada; 7 Division of Adolescent Medicine, Department of Pediatrics, University of Toronto, 555 University Avenue, Toronto, ON, M5G 1X8, Canada; 8 College of Physicians and Surgeons of Ontario, 80 College Street, Toronto, ON, M5G 2E2, Canada; 9 Centre for Addiction and Mental Health, Faculty of Pharmacy and Department of Psychiatry, University of Toronto, 33 Russell Street, Toronto, ON, M5S 2S1, Canada; 10 Faculty of Medicine, University of Ottawa, 451 Smyth Road, Ottawa, ON, K1H 8M5, Canada; 11 Department of Epidemiology & Community Medicine, Faculty of Medicine, University of Ottawa, 451 Smyth Road, Ottawa, ON, K1H 8M5, Canada

**Keywords:** Brief intervention, Drug use, Psychoactive substance, Referral to treatment, SBIRT, Screening, Substance use, Systematic review

## Abstract

**Background:**

There is a significant public health burden associated with substance use in Canada. The early detection and/or treatment of risky substance use has the potential to dramatically improve outcomes for those who experience harms from the non-medical use of psychoactive substances, particularly adolescents whose brains are still undergoing development. The Screening, Brief Intervention, and Referral to Treatment model is a comprehensive, integrated approach for the delivery of early intervention and treatment services for individuals experiencing substance use-related harms, as well as those who are at risk of experiencing such harm.

**Methods:**

This article describes the protocol for a systematic review of the effectiveness of brief interventions as part of the Screening, Brief Intervention, and Referral to Treatment model for reducing the non-medical use of psychoactive substances. Studies will be selected in which brief interventions target non-medical psychoactive substance use (excluding alcohol, nicotine, or caffeine) among those 12 years and older who are opportunistically screened and deemed at risk of harms related to psychoactive substance use. We will include one-on-one verbal interventions and exclude non-verbal brief interventions (for example, the provision of information such as a pamphlet or online interventions) and group interventions. Primary, secondary and adverse outcomes of interest are prespecified. Randomized controlled trials will be included; non-randomized controlled trials, controlled before-after studies and interrupted time series designs will be considered in the absence of randomized controlled trials. We will search several bibliographic databases (for example, MEDLINE, EMBASE, CINAHL, PsycINFO, CORK) and search sources for grey literature. We will meta-analyze studies where possible. We will conduct subgroup analyses, if possible, according to drug class and intervention setting.

**Discussion:**

This review will provide evidence on the effectiveness of brief interventions as part of the Screening, Brief Intervention, and Referral to Treatment protocol aimed at the non-medical use of psychoactive substances and may provide guidance as to where future research might be most beneficial.

## Background

We used the Preferred Reporting Items for Systematic Reviews and Meta-Analyses for Protocols (PRISMA-P) checklist to guide the reporting of this protocol [1]. According to the 2010 Canadian Alcohol and Drug Use Monitoring Survey [2], 11.2% of Canadians aged 15 years and older reported past-year use of at least one of the following psychoactive substances: cannabis, cocaine/crack, methamphetamine/crystal methamphetamine, ecstasy, hallucinogens, salvia, inhalants, heroin, pain relievers, stimulants or sedatives. The rate of past-year use of any of these substances was higher among males than females (15.3% versus 7.5%, respectively). Rates were also higher among those aged 15- to 24-years-old compared with adults 25 years and older (26.3% versus 8.3%, respectively). Furthermore, among those reporting past-year use, 17% reported experiencing substance use-related harm.

There is also a significant public health burden associated with substance use in Canada. According to 2002 estimates, substance abuse costs Canadians close to $40 billion ($1,267 per Canadian), with use of psychoactive substances (excluding alcohol) accounting for approximately $8.2 billion (20.7%) of the total costs [3]. The vast majority of those costs are associated with Canadians’ lost productivity and health.

For the purpose of this review, non-medical psychoactive substance use includes the use of drugs prohibited by international law including, but not limited to, amphetamine-type stimulants, cannabis, cocaine, heroin and 3,4-methylenedioxymethamphetamine (MDMA) [4]; the non-medical use of pharmaceuticals such benzodiazepines, opioids or dextromethorphan; and the use of substances such as solvents or inhalants (for example, gasoline, acetone) when they are used for their intoxicating effects. It does not include alcohol, nicotine or caffeine.

The early detection and/or treatment of risky substance use has the potential to dramatically improve outcomes for those who experience harms from the non-medical use of psychoactive substances, particularly adolescents whose brains are still undergoing development. Screening, Brief Intervention, and Referral to Treatment (SBIRT) is a comprehensive, integrated approach to the delivery of early intervention and treatment services for individuals experiencing substance use-related harms, as well as those who are at risk of experiencing such harms [5]. The SBIRT model is based on public health principles and procedures, and is designed to reduce the burden of injury, disease and disability associated with the non-medical use of psychoactive substances.

The protocol typically begins with a screening procedure that involves asking questions to evaluate whether the individual has experienced, or is at risk of experiencing, substance use-related harms. Brief interventions (BIs) are typically delivered to those individuals at low to moderate risk of harms; individuals identified as experiencing significant harm and/or having more serious signs of substance dependence warranting formal diagnosis may be referred to treatment services that are outside the scope of BIs.

### Screening

To evaluate the likelihood that an individual is experiencing, or is at risk of experiencing, substance use-related harms, individuals are screened. Screening may be conducted in a number of different ways. For example, screening may be conducted via psychometrically validated questionnaires or tests developed to accurately categorize users into low, moderate, and high risk categories. Such tests have been developed for different types of substances such as alcohol (Alcohol Use Disorders Identification Test [6]), cannabis (the Cannabis Use Disorders Identification Test [7]) or prescription opioids (for example, the Opioid Risk Tool [8]). General drug screening tests also exist (for example, the Drug Abuse Screening Test [9]). However, screening tests that reliably categorize users into low or moderate risk groups have not been developed for other substances (for example, heroin and cocaine). For these substances, screening may simply take the form of self-reported use or biological markers indicating use (such as hair, urine, oral fluid or blood) rather than psychometrically validated self-report instruments. In the absence of validated tests or biological markers, others may rely on even less rigorous screening methods, such as the subjective judgment of the individual conducting the assessment. Regardless of the screening method employed, those deemed at risk of harms are typically provided a BI or referred to treatment. In cases where self-reported use or biological markers indicating use are used for screening, it is unclear how practitioners make the decision whether to administer a BI versus referral to treatment.

In reviews where the effectiveness of SBIRT models in reducing harms associated with alcohol use have been evaluated systematically [10-12], few protocols have employed a rigid definition of the screening criteria used to determine whether a BI was administered. This is likely a result of two factors: poor descriptions of screening procedures employed in studies reviewed and/or the heterogeneity of screening procedures employed.

### Brief intervention

In addition to the variability in screening procedures employed, there is also much variation in how BIs are defined and delivered. In general, BIs are in-person, time-limited efforts to provide information or advice, increase motivation to avoid substance use, or to teach behavior change skills with the aim of reducing substance use and the likelihood of experiencing negative consequences. This variation includes the number of conversations or meetings that take place during intervention delivery as well as the amount of time spent conducting the BI. Reviews such as Kaner *et al*. [11] have defined ‘brief’ to mean four or less and note that BIs for alcohol that are provided in primary health care settings are typically delivered within the normal consultation period of 5 to 30 minutes. Levy *et al*. [13] suggest that successful BIs typically focus on the following elements (collectively referred to using the acronym FRAMES): feedback on behavior and consequences; responsibility to change; advice; menu of options to bring about change; empathy; and self-efficacy for change.

There is substantial scientific evidence of the benefits of the SBIRT model in primary health care settings as a means to address the harms associated with alcohol use [14-16]. This evidence suggests the SBIRT process can serve as an effective ‘early warning’ system to prevent and/or reduce the serious long-term harms associated with excessive alcohol use. A corresponding analysis for SBIRT targeting the non-medical use of other psychoactive substances is needed.

There is accumulating evidence suggesting that BIs may be effective in reducing the non-medical use of psychoactive substances, such as cannabis [17-21], ecstasy [22], cocaine [18,23,24], benzodiazepines [25] and opioids [6,13,23] among both youths and adults. Traditionally, the SBIRT model has been implemented in primary care settings, emergency departments, inpatient trauma units and other health care settings. These settings see the broadest number and range of patients and thus provide ideal opportunities to screen for, and address, substance use before more severe consequences occur [26]. More recently, however, the protocol is being applied in schools [21,27] and community settings [22] in an attempt to reach young people. It is unclear whether the effectiveness of the SBIRT approach is dependent on the setting in which it is applied.

The diversity of substances used and the high prevalence of use and dependence have raised some concerns about the efficacy of a SBIRT protocol for substances other than alcohol [26]. Individuals who use more than one substance or use alcohol and other substances make administering and evaluating SBIRT more complicated than when addressing alcohol alone [28]. Substances have variable forms, costs, risks, consequences and ways for clinicians to identify use. Moreover, most psychoactive substances that are used without medical supervision are illegal or used illegally, which can complicate addressing their use in medical settings by raising patient and physician concerns about confidentiality. Prescription drug misuse presents additional challenges as clinicians struggle to distinguish between appropriate and inappropriate use.

### Objectives

This systematic review will determine the effects of BIs, as part of the SBIRT protocol, on reducing substance use in adolescents and adults identified as experiencing, or at risk of experiencing, harms related to the non-medical use of psychoactive substances (excluding alcohol, caffeine and nicotine). In addition, potential moderating factors that may impact SBIRT effectiveness will be evaluated, if possible.

## Methods

### Selection criteria

#### Population

Studies in which BIs are administered to adolescents (12 to 18 years of age or equivalent by level of schooling), young adults (19 to 24 years of age), or adults (25 years and older) at risk of harms related to psychoactive substance use as determined by the screening component of the SBIRT protocol will be included. We will exclude studies evaluating interventions targeting children less than 12 years of age.

#### Screening

Screening must be opportunistic in nature (that is, it must be conducted among a population not seeking treatment for substance use) and is defined as any procedure or method used to identify those experiencing or at risk of harms associated with the non-medical use of psychoactive substances. This may include, but is not limited to, questions regarding the use of substances, the use of psychometrically validated scales, or biological (for example, blood, hair, urine, oral fluid) screening tools. We will exclude studies failing to indicate they used a method of screening to determine who receives a BI.

#### Brief intervention

BIs are time-limited efforts to provide information or advice, increase motivation to avoid substance use, or to teach behavioral change skills with the aim of reducing substance use and the likelihood of experiencing negative consequences.

We will include studies in which BIs target non-medical psychoactive substance use. This includes the use of drugs prohibited by international law including, but not limited to: amphetamine-type stimulants, cannabis, cocaine, heroin and MDMA [4]; the non-medical use of pharmaceuticals such benzodiazepines, opioids or dextromethorphan; and the use of substances such as solvents or inhalants (for example, gasoline, acetone) when they are used for their intoxicating effects. We will exclude studies in which the BI only targets alcohol, nicotine or caffeine use. In addition, interventions that do not provide feedback on at least one of the FRAMES elements or provide five or more sessions [11] will be excluded.

For the current review we will only examine one-on-one verbal interventions and exclude non-verbal BIs (for example, the provision of information such as a pamphlet or online interventions) and group interventions, as these interventions differ sufficiently from verbal one-on-one. However, we will collate studies assessing the effectiveness of online and group interventions and report on the search yield in the review.

#### Referral to treatment

We will not address the effectiveness of the referral to treatment component of the SBIRT protocol in this review.

#### Comparisons

Studies comparing BI to no BI, provision of pamphlets or other information only, or delayed intervention will be included. Studies without a comparison or control group will be excluded.

#### Outcomes

We will evaluate the outcomes listed below for any period of follow-up.

Primary outcomes:

1. Substance use versus non-use.

2. Frequency and quantity of use.

3. Any standard or accepted biological markers of substance use (for example, blood, oral fluid).

4. Self- or other reported use-related harms or negative consequences of use.

5. Self- or other reported changes in behavior likely to result in the reduction of negative substance use-related consequences.

6. Decision to attend treatment.

Secondary outcomes:

1. Use of different substances from that for which the client received the BI (including use of alcohol, caffeine, or nicotine) as assessed by self- or other reported or biological markers substance use.

2. Self- or other reported intention to reduce substance use.

3. Self- or other reported health measure.

Adverse outcomes:

1. Self- or other reported use or increased use of different substances from that for which the client received the BI as assessed by self- or other reported or biological markers substance use.

2. Other adverse outcomes.

#### Settings

We will include studies that use opportunistic screening regardless of location, including, but not limited to, primary health care, emergency room, school and other community settings.

#### Study designs

All randomized controlled trials (RCTs) will be included, including any cluster RCTs. Where RCTs are lacking or, for issues relating to feasibility, not conducted, non-RCTs, controlled before-after studies and interrupted time series designs will be considered. These latter designs will include some form of control group, whether concurrent or within-patient. Interrupted time series studies must have a clearly defined time point at which the intervention is introduced and at least three data points before and after introducing the intervention; those ignoring secular changes and performing simple pre-post analyses will not be included unless re-analysis is possible [29]. We will not evaluate retrospective studies or studies with historical controls. We will exclude comments, letters and editorials.

### Search methods

#### Electronic bibliographic databases

A comprehensive literature search for studies and systematic reviews using high recall subject searches will be conducted using several electronic databases: MEDLINE, EMBASE, *The Cochrane Library*, Cumulative Index to Nursing and Allied Health Literature (CINAHL), PsycINFO, Education Resources Information Center (ERIC) and the CORK Database. All electronic search strategies will be peer-reviewed using the PRESS tool prior to implementation [30]. The proposed MEDLINE search strategy is shown in Appendix 1; the search will be adapted to the other databases.

#### Other sources

‘Grey literature’ searches will be conducted for other potentially relevant studies. Websites of health technology assessment and evidence-based review organizations as listed in *Grey Matters: a practical tool for evidence-based searching*[31] will be searched as well as PsycEXTRA. Websites of relevant organizations, such as the Canadian Centre on Substance Abuse, Centre for Addiction and Mental Health, the Substance Abuse and Mental Health Services Administration, National Institute for Drug Abuse and the Centre for Addictions Research British Columbia, as well as those indicated on the Substance Abuse Librarians & Information Specialists site will be searched. We will also access the websites of specific organizations, such as the College of Physicians and Surgeons of Ontario. We will consult within our internal team to identify additional studies that may not be in the published literature. We will search the Campbell Collaboration for systematic reviews. We will scan bibliographies of included articles and relevant systematic reviews. We will consult clinicaltrials.gov and the WHO International Clinical Trials Registry Platform for ongoing studies.

#### Language

We will not restrict our search based on language but will only include articles available in the English and French languages. A list of the possibly relevant titles available in other languages will be provided as an appendix.

#### Study selection

The results of the literature search will be assessed using a two-step process. First, one individual will screen citations by title and/or abstract according to the prespecified screening questions (level 1). Those records deemed to be ‘included’ or ‘unclear’ will automatically pass to the next level of screening (level 2). However, if the record is deemed ‘excluded’, then it needs to be reviewed by a second reviewer to confirm exclusion. This process is referred to as liberal accelerated screening, a more efficient means of initially assessing records for relevancy.

Second, full text screening will be performed independently by two reviewers and discordance will be resolved first by consensus and then by third member of the research team, as needed (level 2 assessment). The selection process and reasons for exclusion will be documented using the PRISMA flow diagram [32].

Literature search results will be uploaded to Distiller Systematic Review Software (DSR), an Internet-based software program that facilitates collaboration among reviewers during the study selection process. The team will develop and test screening questions and forms for level 1 and 2 assessments based on the inclusion and exclusion criteria. Citation abstracts and full text articles will be uploaded with screening questions to DSR. Prior to the formal screening process, a calibration exercise will be undertaken to pilot and refine the screening questions. Further, we will provide training to new members of the review team not familiar with the DSR software and the content area prior to the start of the review.

### Data abstraction

Two independent reviewers will extract and document the content of each included study using a standardized data abstraction form in DSR to capture information on the descriptive and quantitative characteristics of individual studies. Useful data to collect will include:

1. publication details (for example, year of publication, language of publication, country in which the study was conducted, publication status, sources of funding);

2. substance targeted by the BI;

3. study details (for example, date, number of centers, follow-up);

4. study design methodology (for example, methods of allocation, blinding) for conducting a risk of bias assessment;

5. population details of the target group (for example, sample size, characteristics such as age, sex and ethnic background);

6. details on the screening method used to determine who should be provided a BI (for example, nature of method, whether validated, administration method, length to screen, type of professional conducting the screening, the extent of training they received in conducting screening);

7. details of the intervention and comparison groups, including any theoretical frameworks and intensity of the intervention (for example, length of and time between sessions);

8. fidelity of the intervention;

9. setting;

10. outcome details including definitions, outcome measures used and data.

Disagreements will be resolved first by consensus and then by a third member of the research team, as needed. Prior to performing data abstraction, the review team will refine the development of the extraction forms and will conduct a calibration and training exercise.

### Assessing the risk of bias

Two independent reviewers will assess the risk of bias for each included study. Any disagreements will be resolved first through discussion and then by third member of the research team, as needed. Determining risk of bias summary assessments for outcomes will follow that proposed by The Cochrane Collaboration [33] and incorporated into grading the quality of evidence.

All RCTs will be assessed using the Cochrane Risk of Bias (RoB) tool [33]. The Cochrane RoB tool evaluates seven domains (sequence generation, allocation concealment, blinding of participants and personnel, blinding of outcome assessors, incomplete outcome data, selective outcome reporting, and other sources of bias). Other sources of bias will include single versus multicenter studies and study sponsorship. We will also assess cluster randomized trials for the possibility of recruitment bias [34]. If included, other study designs will be critically appraised with the Cochrane Effective Practice and Organisation of Care group’s modified RoB tool (Appendix 2) [29].

### Unit of analysis issues

For studies whose allocation design is a group level (for example, schools) and yet analyzed at the individual level (for example, students), ‘unit of analysis’ errors can occur, whereby results can be overly precise and contribute greater weight in a meta-analysis. Where possible, we will adjust the analysis according to the intracluster correlation coefficient to address these errors [35], preferably with empirically-derived values.

### Missing data

If information or data is missing or incomplete, we will attempt to contact the study authors twice over four weeks by email. We will analyze data as close to the intention-to-treat principle (all participants analyzed according to assigned treatment group) as possible. If loss to follow-up occurs, we will incorporate any additional data provided in study reports or by the authors; we will not impute data for any outcomes.

### Evidence synthesis

Study characteristics will be summarized narratively in the text, and/or summary tables in the report; such data may be presented as frequencies and percentages, medians and interquartile ranges, or means and standard deviations, where appropriate. For outcomes, where appropriate and possible (depending on the quantity, quality, and statistical and clinical homogeneity of the available data), pooled estimates of intervention efficacy or effectiveness will be computed using standard meta-analytic methods [36]. Random effects models will be used for all meta-analyses. A narrative synthesis of the evidence will be conducted when quantitative pooling of data is not possible.

#### Main analyses

For the primary analysis, our intent is to evaluate all BIs for all drug classes together. We are uncertain what exists in the literature regarding how interventions are targeted (that is, for general drug use or for specific drugs), and we do not know how varied they may be in study characteristics or methodology. Before proceeding with a pooled analysis, we will determine whether studies are homogeneous with respect to those characteristics. If too much heterogeneity exists, we will not meta-analyze all studies together. In addition, should considerable statistical heterogeneity (≥75%) exist, we will also not present a pooled analysis.

We will also conduct subgroup analyses according to drug class (for example, cannabinoids, opioids and amphetamines, with general use as a separate category) and intervention setting (for example, school, hospital, community).

#### Dichotomous outcomes

For dichotomous outcomes, the risk ratio and 95% confidence intervals will be presented. The unit of analysis is the proportion of study participants with the outcome for intervention and control groups.

#### Continuous outcomes

For continuous outcomes, mean differences and 95% confidence intervals will be used for outcomes reported on similar scales or with similar measures; standardized mean differences will be used where continuous outcomes are reported using different scales or measures. Transformation of data to allow analyses with mean differences will be made wherever possible. Where change-from-baseline data are reported in the studies, we will extract in addition to post-intervention data.

#### Ordinal outcomes

Decisions about handling and analyzing ordinal outcomes will be determined post hoc, subject to the body of evidence available.

#### Time-to-event data

For time-to-event data, the generic inverse variance method will be used to meta-analyze outcomes using log hazard ratios and standard errors; the Parmar method [37] will be used to estimate data, as needed.

#### Other statistical considerations

##### Data conversions

Where needed, we will convert data (for example, standard error to standard deviation) for use in analyses and to facilitate consistent presentation of results across studies.

##### Interrupted time series designs

If interrupted time series studies are included we will re-analyze data, where needed and if feasible, for change in level and slope according to time series regression analyses [38].

#### Statistical heterogeneity

Statistical heterogeneity will be evaluated using I-squared (I^2^) statistics; for the interpretation of I^2^, a rough guide of low (0% to 25%), moderate (25% to 50%), substantial (50% to 75%), and considerable (75% to 100%) heterogeneity will be used [36,38]. Possible reasons contributing to heterogeneity will be explored.

#### Exploring statistical heterogeneity

If studies are determined to be statistically heterogeneous, we will conduct the following subgroup analyses, where possible:

1. age of participants (12 to 18 years, 19 to 24 years, 25 years or greater);

2. sex;

3. description of screening method (well-described, poorly described, unclear screening method);

4. type of intervention provided (for example, cognitive behavioral therapy, motivational enhancement therapy, motivational interviewing);

5. type of practitioner.

If appropriate and with sufficient, complete data, we will verify results of the subgroup analyses with univariable meta-regression. The variables outlined above for subgroup analyses will be considered statistically significant at *P* <0.01.

#### Sensitivity analyses

If data permit, and where needed, sensitivity analyses may be undertaken regarding the risk of bias (restricting the analyses to studies with low risk of bias), the fidelity of the intervention, data issues and measurement of outcomes or to explore reasons for heterogeneity.

#### Test for funnel plot asymmetry

If at least 10 studies are included in a given meta-analysis, we will evaluate for funnel plot asymmetry, depending on the outcome, and postulate reasons for the asymmetry (for example, publication bias) [39-41].

### Grading the quality of evidence

The quality of evidence for all outcomes will be judged using the Grading of Recommendations Assessment, Development and Evaluation working group methodology [41]. The quality of evidence will be assessed across the domains of risk of bias, consistency, directness, precision and publication bias. Additional domains may be considered where appropriate. Quality will be adjudicated as high (further research is very unlikely to change our confidence in the estimate of effect), moderate (further research is likely to have an important impact on our confidence in the estimate of effect and may change the estimate), low (further research is very likely to have an important impact on our confidence in the estimate of effect and is likely to change the estimate), or very low (very uncertain about the estimate of effect).

### Quality assurance

We will discuss results in light of the strength of findings as well as their research and practice implications. This review will be reported according to the PRISMA statement [32] and using the assessment of multiple systematic reviews tool for additional quality control [42].

## Discussion

The accumulating research assessing the effectiveness of SBIRT for the non-medical use of psychoactive substances underscores the need for a systematic review in order to assist clinicians and others to inform evidence-based practice. The results from a recent evidence map of systematic reviews to inform the prevention, treatment and/or harm reduction for illicit drug use [43] revealed no published systematic reviews or meta-analyses on the effectiveness of the SBIRT model in reducing illicit drug use. A systematic review will yield a better understanding of the effectiveness of BIs as part of SBIRT aimed at the non-medical use of psychoactive substances, which will be helpful in establishing guidelines for implementation in general practice and other relevant settings. Finally, synthesizing the evidence base may provide guidance as to where future research might be most beneficial.

## Abbreviations

BI, Brief intervention; CINAHL, Cumulative Index to Nursing and Allied Health Literature; DSR, Distiller Systematic Review Software; ERIC, Education Resources Information Center; FRAMES, Feedback on behavior and consequences, Responsibility to change, Advice, Menu options to bring about change, Empathy, Self-efficacy for change; MDMA, 3:4-methylenedioxymethamphetamine; PRISMA-P, Preferred Reporting Items for Systematic Reviews and Meta-Analyses for protocols; RCT, Randomized control trial; RoB, Risk of bias; SBIRT, Screening, Brief Intervention, and Referral to Treatment.

## Competing interests

The authors declare that they have no competing interests.

## Authors’ contributions

MMY is the guarantor. MMY, AS, APW, TP and CG drafted the manuscript. MMY, AS and TP developed the selection criteria, specified outcome measures and determined the data to be abstracted. AS, LT and MMY determined the analyses to be conducted. CG, APW and BS developed the search criteria. All authors read, provided feedback and approved the final manuscript.

## Appendices

### Appendix 1. Proposed MEDLINE search strategy

1.SBIRT.tw.

2. (SBI or SBIs).tw.

3. exp Substance Abuse Detection/

4. ((substance* adj2 test*) or (substance* adj2 detect*) or (substance* adj2 screen*) or (drug* adj2 detect*) or (drug* adj2 screen*) or (drug* adj2 test*)).tw.

5. 3 or 4

6. Substance-Related Disorders/

7. exp Amphetamine-Related Disorders/

8. exp Cocaine-Related Disorders/

9. exp Marijuana Abuse/or exp Marijuana Smoking/

10. exp Opioid-Related Disorders/

11. exp Phencyclidine Abuse/

12. Psychoses, Substance-Induced/

13. exp Substance Abuse, Intravenous/

14. ((substance-related or substance-induced) adj3 (disorder* or psychosis or psychoses)).tw.

15. ((drug or drugs or substance* or opioid* or opiate* or amphetamine* or amfetamine* or methamphetamine* or methamfetamine or benzodiazepine* or morphine* or methadone* or prescription* or phencyclidine* or solvent* or barbiturate* or depressant* or stimulant* or psychotherap* or psycho-therap* or steroid*) adj3 (addict* or abuse* or abusing or abusive or misuse* or mis-use* or misusing or mis-using or illicit* or illegal* or unlawful* or unsanction* or habit* or dependen* or disorder or disorders or relapse* or consumption)).tw.

16. or/6-15

17. Dextropropoxyphene/

18. (Dextropropoxyphene or D-Propoxyphene or Propoxyphene or Darvon or Vicodin).tw.

19. Hydromorphone/

20. (Hydromorphon* or Dihydromorphinone or Dilaudid or Laudacon or Palladone).tw.

21. exp Meperidine/

22. (Meperidine or Demerol or Dolantin or Dolargan or Dolcontral or Dolin or Dolosal or Dolsin or Isonipecain or Lidol or Lydol or Operidine or Pethidine or Promedol or Dimethylmeperidine or Isopromedol or Trimeperidine or Lomotil or Reasec).tw.

23. Pentobarbital/

24. (Pentobarbital or Diabutal or Etaminal or Ethaminal or Mebubarbital or Mebumal or Nembutal or Pentobarbitone or Sagatal).tw.

25. exp Diazepam/

26. (Diazepam or Apaurin or Diazemuls or Faustan or Relanium or Seduxen or Sibazon or Stesolid or Valium or Nordazepam or Calmday or Dealkylprazepam or Demethyldiazepam or Deoxydemoxepam or Desmethyldiazepam or Nordaz or Nordiazepam or Norprazepam or "Tranxilium N" or Vegesan).tw.

27. ("substance use" or "substance usage").tw.

28. Alprazolam/

29. (Alprazolam or Alprazolan or Alprox or "Apo-Alpraz" or Cassadan or Esparon or Kalma or "Novo-Alprazol" or "Nu-Alpraz" or Ralozam or Tafil or Trankimazin or Xanax).tw.

30. Dextroamphetamine/

31. (Dextroamphetamine or Curban or "d-Amphetamine" or Dexamfetamine or Dexamphetamine or Dexedrine or dextro-Amphetamine or DextroStat or Oxydess).tw.

32. Methylphenidate/

33. (Methylphenidate or Centedrin or Daytrana or Dexmethylphenidate or Equasym or Focalin or Metadate or Methylin or Phenidylate or Ritalin* or Tsentedrin or Adderall or Obetrol).tw.

34. or/17-33

35. 34 and (addict* or abuse* or abusing or abusive or misuse* or mis-use* or misusing or mis-using or illicit* or illegal* or unlawful* or unsanction*).tw.

36. 16 or 35

37. exp designer drugs/or exp street drugs/

38. (designer drug$1 or street drug$1 or recreational drug$1 or narcotic* or non-therapeutic drug$1 or non-medical drug$1).tw.

39. exp Cannabis/

40. (Cannabi* or marijuana or marihuana or hemp or hash or hashish or ganja).tw.

41. exp Heroin/

42. (Heroin or Diacetylmorphine or Diagesil or Diamorphine or Diamorf or speed).tw.

43. exp Cocaine/

44. (cocaine or crack).tw.

45. exp Hallucinogens/

46. (hallucinogen* or psychedelic*).tw.

47. exp amphetamine/or exp methamphetamine/or exp n-methyl-3,4-methylenedioxyamphetamine/or mescaline/or ketamine/or oxycodone/

48. (methylenedioxymethamphetamine or MDMA or ecstasy or methamphetamine or crystal meth or mescaline or mezcalin or peyote or trimethoxyphenethylamine or ketamine or Calipsol or Calypsol or "CI-581" or Kalipsol or Ketalar or Ketanest or Ketaset or "special k" or mushroom* or prilocybin or oxycodone* or oxycontin* or dihydrohydroxycodeinone or dihydrone or dinarkon or eucodal or oxiconum or oxycodeinon or oxycone or pancodine or theocodin or "gamma hydroxybutrate" or GHB or PCP).tw.

49. ((sniff* or inhal* or snort*) adj3 (solvent* or glue or drug or drugs)).tw.

50. ("inhalant use" or "inhalant usage").tw.

51. exp Lysergic Acid Diethylamide/

52. (LSD or Lysergide or Lysergic Acid Diethylamide).tw.

53. ("drug use" or "drug usage" or (drug adj user*)).tw.

54. or/37-53

55. 36 or 54

56. Mass Screening/

57. screen*.tw.

58. Self Disclosure/

59. (detect* or disclos* or identif* or question* or reveal* or survey* or unveil*).tw.

60. Interview, Psychological/

61. ((interview* or encounter* or visit*) adj3 (counsellor* or counselor* or nurse* or physician* or doctor* or clinician* or psychologi* or social worker*)).tw.

62. or/56-61

63. 55 and 62

64. 5 or 63

65. Psychotherapy, Brief/or Crisis Intervention/

66. (brief intervention* or brief prevention*).tw.

67. 65 or 66

68. exp Motivation/

69. exp Psychotherapy/

70. (psycho-therap* or psychotherap*).tw.

71. ((cogniti* or behavior* or behaviour* or conditioning or motivation* or psychosocial* or psycho-social* or psychological) adj3 (therapy or therapies or therapeutic or interven* or modify or modifies or modified or modification)).tw.

72. (counselling or counseling).tw.

73. or/68-72

74. (brief* or short* or short-rang* or short-term or abbreviate* or concise or limited or time-limited or crisis or crises or immediate*).tw.

75. 73 and 74

76. 67 or 75

77. exp "Referral and Consultation"/

78. (refer or refers or referral* or gatekeeper* or gate-keeper* or specialist* or specialty).tw.

79. 77 or 78

80. 64 and (76 or 79)

81. 1 or 2 or 80

82. limit 81 to "reviews (specificity)"

83. meta analysis.pt.

84. exp meta-analysis as topic/

85. (meta-analy* or metanaly* or metaanaly* or met analy* or integrative research or integrative review* or integrative overview* or research integration or research overview* or collaborative review*).ti,ab.

86. (systematic review* or systematic overview* or technology assessment* or HTA or HTAs).ti,ab.

87. exp Technology assessment, biomedical/

88. health technology assessment winchester england.jn.

89. (evidence report technology assessment or evidence report technology assessment summary).jn.

90. or/83-89

91. 81 and 90

92. 82 or 91

93. (controlled clinical trial or randomized controlled trial).pt.

94. exp RCTs as topic/or exp controlled clinical trials as topic/or exp random allocation/or exp double-blind method/or exp single-blind method/or exp placebos/

95. "controlled clinical trial".tw.

96. (random* or RCT$1 or placebo*).tw.

97. ((singl* or doubl* or trebl* or tripl*) and (mask* or blind* or dumm*)).tw.

98. or/93-97

99. 81 and 98

100. clinical trial.pt.

101. exp Clinical Trials as Topic/

102. "clinical trial".ti,ab.

103. (volunteer or volunteers or open label* or nonrandom* or non random* or quasirandom* or quasi-random*).tw.

104. exp Cohort Studies/or exp Longitudinal Studies/or exp Prospective Studies/or exp Follow-Up Studies/

105. (cohort or cohorts or longitudinal or prospective).tw.

106. ((observational or follow-up or followup) adj stud*).tw.

107. (population-based stud* or population-based analys* or population stud* or population analys*).tw.

108. ((descriptive adj stud*) or (multidimensional adj stud*) or (multi-dimensional adj stud*) or (multicenter adj stud*) or (multi-center adj stud*) or (multicentr* adj stud*) or (multi-centr* adj stud*)).tw.

109. exp Multicenter Study/or exp Multicenter Studies as Topic/

110. Comparative Study.pt.

111. ((comparative adj study) or (comparative adj studies) or "before and after").tw.

112. or/100-111

113. 81 and 111

114. 92 or 99 or 113

### Appendix 2. Cochrane Effective Practice and Organisation of Care Review group’s criteria for assessing risk of bias in non-randomized, controlled before-and-after, and interrupted time series studies

#### Non-randomized controlled trials and controlled before-after studies

· Was the allocation sequence adequately generated?

· Was the allocation adequately concealed?

· Were baseline outcome measurements similar?

· Were baseline characteristics similar?

· Were incomplete outcome data adequately addressed?

· Was knowledge of the allocated interventions adequately prevented during the study?

· Was the study adequately protected against contamination?

· Was the study free from selective outcome reporting?

· Was the study free from other risks of bias?

#### Interrupted time series studies

· Was the intervention independent of other changes?

· Was the shape of the intervention effect prespecified?

· Was the intervention unlikely to affect data collection?

· Was knowledge of the allocated interventions adequately prevented during the study?

· Were incomplete outcome data adequately addressed?

· Was the study free from selective outcome reporting?

· Was the study free from other risks of bias?
